# Associations between implementation of Project Lazarus and opioid analgesic dispensing and buprenorphine utilization in North Carolina, 2009–2014

**DOI:** 10.1186/s40621-018-0179-2

**Published:** 2019-01-21

**Authors:** Apostolos A. Alexandridis, Nabarun Dasgupta, Agnieszka D. McCort, Christopher L. Ringwalt, Wayne D. Rosamond, Paul R. Chelminski, Stephen W. Marshall

**Affiliations:** 1 Injury Prevention Research Center, University of North Carolina at Chapel Hill, Chapel Hill, North Carolina USA; 2 Department of Epidemiology, Gillings School of Global Public Health, University of North Carolina at Chapel Hill, Chapel Hill, North Carolina USA; 3 Department of Medicine, School of Medicine, University of North Carolina at Chapel Hill, Chapel Hill, North Carolina USA

**Keywords:** Opioids, Overdose, Buprenorphine, MAT, Community coalitions, Prevention, PDMP, Interrupted time series, Evaluation

## Abstract

**Background:**

Project Lazarus (PL) is a seven-strategy, community-coalition-based intervention designed to reduce opioid overdose and dependence. The seven strategies include: community education, provider education, hospital emergency department policy change, diversion control, support programs for patients with pain, naloxone policies, and addiction treatment expansion. PL was originally developed in Wilkes County, NC. It was made available to all counties in North Carolina starting in March 2013 with funding of up to $34,400 per county per year. We examined the association between PL implementation and 1) overall dispensing rate of opioid analgesics, and 2) utilization of buprenorphine. Buprenorphine is often used in connection with medication assisted treatment (MAT) for opioid dependence.

**Methods:**

Observational interrupted time series analysis of 100 counties over 2009–2014 (*n* = 7200 county-months) in North Carolina. The intervention period was March 2013–December 2014. 74 of 100 counties implemented the intervention. Exposure data sources comprised process surveys, training records, Prescription Drug Monitoring Program (PDMP) data, and methadone treatment program quality data. Outcomes were PDMP-derived counts of opioid prescriptions and buprenorphine patients. Incidence Rate Ratios were estimated with adjusted GEE Poisson regression models of all seven PL strategies.

**Results:**

In adjusted models, diversion control efforts were positively associated with increased dispensing of opioid analgesics (IRR: 1.06; 95% CI: 1.03, 1.09). None of the other PL strategies were associated with reduced prescribing of opioid analgesics. Support programs for patients with pain were associated with a non-significant decrease in buprenorphine utilization (IRR: 0.93; 95% CI: 0.85, 1.02), but addiction treatment expansion efforts were associated with no change in buprenorphine utilization (IRR: 0.98; 95% CI: 0.91, 1.06).

**Conclusions:**

Implementation of PL strategies did not appreciably reduce opioid dispensing and did not increase buprenorphine utilization. These results are consistent with previous findings of limited impact of PL strategies on overdose morbidity and mortality. Future studies should analyze the uptake of MAT using a more expansive view of institutional barriers, treating community coalition activity around MAT as an effect modifier.

## Background

Deaths from opioid overdose began increasing in North Carolina (NC) in the late 1990s (Web-based Injury Statistics Query and Reporting System (WISQARS), 2005). Between 1999 and 2015, opioid mortality increased 486% to over 11 per 100,000 (Injury and Epidemiology Surveillance Unit, Injury and Violence Prevention Branch, Division of Public Health, North Carolina Department of Health and Human Services, 2015). Opioid overdose has become the leading cause of unintentional injury death in the state, and involves prescription opioid analgesics (OA) as well as illicitly manufactured heroin and fentanyl (State Center for Health Statistics, 2015). Addressing this epidemic has become a leading priority for the NC Department of Health and Human Services (NC DHHS), which has promoted supply, demand, and harm reduction strategies (North Carolina Department of Health and Human Services, 2017).

Among demand reduction strategies, medication assisted treatment (MAT), particularly with the partial opioid agonist buprenorphine, has been widely embraced. MAT is supported by substantial evidence-based modalities of substance abuse treatment (Mattick et al., 2014; Thomas et al., 2014). Buprenorphine is the only form of agonist MAT that can be dispensed by traditional retail pharmacies, and can be prescribed by primary care providers who complete an 8-h training through the Substance Abuse and Mental Health Services Administration (SAMHSA) (Fiellin et al., 2004). Buprenorphine also has advantages for patients seeking agonist-based MAT in rural areas (Kraus et al., 2011). Formulations of buprenorphine indicated for MAT are also often used to reduce the risk of opioid abuse in patients receiving high doses of full-agonist opioids.

Project Lazarus (PL) is a comprehensive, community-based series of seven interventions designed to reduce demand, supply and harms related to prescription OA; improve treatment of chronic pain; and promote and improve access to MAT. PL was first piloted in one NC county between 2007 and 2010, and was implemented statewide in early 2013 (Albert et al., 2011). Subsequently, the seven distinct PL strategies were promoted nationally by the White House Office of National Drug Control Policy (ONDCP) 2015 opioid strategy (United States, 2015). Funding for coalitions was made available to all 100 NC counties through a non-competitive application process organized by the state Medicaid implementation authority, Community Care of North Carolina (CCNC), and the Mountain Area Health Education Center (MAHEC). Coalitions were invited to select among the seven PL strategies they felt best represented their community’s needs, with a minimum of three.

PL’s seven distinct strategies are designed to be implemented together by a community-based coalition. This paper examines the association between the seven PL strategies and (1) overall prescribing rate of opioid analgesics and (2) utilization of buprenorphine. The 7 PL strategies are as follows. (1) *Community education* promoted public awareness of prescription opioid overdose. (2) *Diversion control* was designed to remove unused medications and train law enforcement on OA diversion. (3) *Support programs for patients with pain* provided support groups, case management and pain clinic vetting and referrals. (4) *Provider education* focused on educating medical professionals in chronic pain treatment, including group trainings and in-office ‘academic detailing,’ or tailored instruction. The North Carolina Medical Board’s published guidelines for pain management were referenced in trainings (Trado, 2004). (5) *Hospital emergency department (ED) policies* revised hospital practices to limit ED OA prescribing and require checking the state’s Prescription Drug Monitoring Program (PDMP) before prescribing. (6) *Addiction treatment* expansions increased the number of providers in a community able to prescribe buprenorphine-based MAT for opioid dependence, and the number of beds available in inpatient detoxification and treatment facilities. (7) *Naloxone policies* promoted liberal distribution of the opioid antagonist naloxone to opioid users and their close contacts, first responders including EMS and police, and caregivers. Strategies 1–3 were focused on community entities external to the health care system, whereas strategies 4–7 were focused on health care providers (Table 1).

**Table 1:** Project Lazarus strategies and hypothesized effects

	Strategy	Examples of Key Activities	Expected effect on opioid analgesic prescribing	Expected effect on buprenorphine utilization
1	Community Education	• Community- and school-based prevention education	Decrease	Increase
		• Multi-media advertising campaigns		
2	Diversion Control	• Education on proper storage of medications	No change	No change
		• Pill take-backs and fixed disposal sites		
		• Training of law enforcement in the prevention of medication diversion and the arrest of diverters		
3	Support for Patients with Pain	• Development of support groups and other extra-clinical services for patients	Decrease	Increase
		• Referrals of patients to clinics		
		• Pain patient education on reducing risks of overdose		
4	Provider Education	• Continuing education on the effective management of chronic pain and appropriate opioid prescribing	Greatest decrease	Increase
		• Effective management of patients with chronic pain		
5	Hospital Emergency Department (ED) Policy	• Policies to limit controlled substance dispensing in EDs	Decrease	No change
		• Policies to require use of the PDMP in EDs		
6	Addiction Treatment	• Increases in availability/access to drug detoxification programs and treatment clinics	No change	Greatest increase
		• Increases in the number of providers authorized to prescribe buprenorphine for addiction		
7	Naloxone Policies	• Provision of naloxone to patients and nonmedical opioid users with high overdose risk and their family members	No change	No change
		• Education on reversing overdose before EMS arrival		

The statewide implementation of PL in NC has significance as one of the earliest and largest coordinated efforts to address the overdose epidemic using community-based approaches. We hypothesized that the seven PL strategies would have varying effects on opioid overdose morbidity and mortality, opioid prescribing, and utilization of buprenorphine (Table 1). This paper focuses on PL’s hypothesized effects on opioid prescribing and buprenorphine utilization. An evaluation of the association between PL and opioid overdose morbidity and mortality has appeared elsewhere (Alexandridis et al., 2018).

## Methods

NC is a large state in the southeastern US (population 9.9 million in 2014) that had overdose rates comparable to the US average during the 2009–2014 study period. We used an interrupted time series design to examine the relationship between strategies implemented as a part of PL and both prescription OA dispensing and buprenorphine utilization rates.

The general analytic approach has been described previously (Alexandridis et al., 2018). Primary and administrative secondary data sources were aggregated at the level of the county for every month over the time period 2009–2014. These secondary data sources included the state PDMP and drug treatment intake interviews. The resulting time series captured relevant activities of PL coalition activities and opioid-related outcomes across a total of 7200 county-months.

### Implementation of PL strategies

PL strategies were implemented by a series of county-based community coalitions. Funding for the intervention was made available to all 100 NC counties via an application process for county-based coalitions beginning in 2011. Funding was distributed through CCNC (the designated state Medicaid implementation authority) and MAHEC, with technical support from the community-based organization Project Lazarus. Coalitions that applied received annual grants of between $6500 and $34,400, from a network of funding sources. Thus, a coalition that received the maximal funding ($34,440) may have been able to provide a full-time salary for a community health worker paid at the average weekly wage in North Carolina. Given that additional coordinators and non-personnel costs would be needed to successfully implement the seven strategies, it is reasonable to assume that no county received funding sufficient to fully implement PL without the need for additional investment by the county or community. Our evaluation included a pre-intervention period (January 2009–February 2013) and an intervention period (March 2013–December 2014). CCNC also funded Medicaid regional coordinators who provided technical assistance to community coalitions, directed provider education, and advocated for changes in hospital policies related to opioid prescribing.

We used measures of coalition activities and ongoing surveys of key community coalition leaders to capture the implementation of the 7 PL strategies in each county in each month. We coded implementation of PL strategies using dichotomous variables that captured the implementation of each strategy, with ‘0’ representing no implementation of a strategy in a county to-date, and ‘1’ representing any ongoing or prior implementation or policy change specific to each strategy.

Community-based coalitions were identified at the time they were funded by CCNC. Coalition activities were captured through structured surveys that three of the authors (ADM, ND, CLR) administered via web survey every 6 months to coalition leaders and the CCNC regional coordinators. Surveys included details on naloxone policy adoption, ED policy changes, creation of support programs for patients with pain, and the location and date of provider and community education events.

For the diversion control strategy, details of the time and location of local law enforcement trainings on diversion control were obtained from the NC State Bureau of Investigation (SBI).

For the addiction treatment strategy and the evaluation of PL association with opioid dispensing, we combined survey data on MAT expansions with measures of incident buprenorphine and methadone utilization. This measure was constructed with data from the NC Controlled Substance Reporting System (CSRS), the state PDMP, and the NC Treatment Outcomes and Program Performance System (NC-TOPPS), a quality monitoring system sponsored by the Substance Abuse and Mental Health Services Administration (SAMHSA). Overall counts of new methadone treatment program patients were abstracted from intake interviews, and added to measures of incident MAT. Buprenorphine treatment episodes were considered incident after a 90-day washout period since the last buprenorphine script dispensed. The evaluation of PL's association with buprenorphine utilization only used the former survey data on MAT expansions and policy change.

### Opioid prescribing and buprenorphine utilization

Data from the CSRS were used to construct county-month counts of patients and prescriptions for opioid analgesics. PDMPs such as the CSRS are state government-run electronic databases that can be queried at the point of care by clinicians to review a patient’s history of receiving controlled substances. Selected law enforcement officers and medical examiners are allowed access to the database when they are investigating specific cases. The CSRS began collecting data in January 2009, and data were provided by the NC Division of Mental Health, Developmental Disabilities, and Substance Abuse Services (DMHDDSAS). The data are generated when prescriptions for controlled substances are dispensed at regulated pharmacies in North Carolina. The data captured comprise each field of information legally required to be included in a North Carolina prescription for a controlled substance. The data are stored locally at the pharmacy and transmitted periodically to a central database. Data elements include unique identifiers for prescribers, dispensers, and patients and their locations; quantity, dose, days supply, and National Drug Code of the prescription; and age and sex of the patient.

The raw data were tabulated by active pharmaceutical ingredient (API) and dosage form (e.g., solid oral, patch) for opioid analgesics. Opioid analgesics were defined as solid oral, transbuccal, or transdermal formulations containing codeine, fentanyl, hydrocodone, hydromorphone, methadone, morphine, oxycodone and oxymorphone. Prescriptions with APIs comprising the top 99.9% of all prescription records were retained; data cleaning removed non-controlled substances and appended metadata on drug class. Patients were assigned a unique identification number provided by the database vendor (Health Information Designs, Auburn, Alabama, USA), which takes name, date of birth, and residential ZIP code into account, and was provided as a one-way hash algorithm and was continuous over data-years.

Data from the CSRS were used to create county-month counts for the two outcome measures of interest, opioid prescribing and buprenorphine utilization. For opioid prescribing, counts of dispensed full mu-opioid receptor (MOR) agonist analgesics and their prodrugs, including solid oral, transdermal, nasal spray, and transbuccal formulations, were extracted by county and month from the CSRS. For buprenorphine utilization, counts of unique monthly buprenorphine patients, created using prescription data from the CSRS, were used to identify all patients receiving pharmacy-dispensed formulations of buprenorphine with indications for addiction treatment (e.g. Subutex, Suboxone, but not Butrans).

### Covariate measures

In order to control for fundamental differences in health status between counties (e.g., “healthy county effect”) and over time, a construct from the Robert Wood Johnson Foundation (RWJF) County Health Rankings was used (Remington et al., 2015). This “county health factors” variable is a composite Z-score-based ranking which comprises health behaviors, including tobacco use, diet and exercise, alcohol and drug use, and sexual activity; clinical care, including access to and quality of care; social and economic factors, including education, employment, income, family and social support, and community safety; and physical environment, including air and water quality, housing and transit. This was available for 2010 onwards; for 2009 the data from 2010 to 2016 were used to linearly extrapolate county months. Annual data were linearly interpolated to generate county-month scores. This score was used in fully-adjusted, immediate-effect multivariable models of all seven strategies.

### Statistical methods

We used incidence rate ratios (IRRs) to quantify the association between the each of the seven PL strategies and our two outcomes, and used Poisson regression to model the IRRs. Models were fit using generalized estimating equations (GEE) to account for clustering at the county level, with a population offset for each county-month (Alexandridis et al., 2018). These models were assessed for indicators of overdispersion, and Negative Binomial (NB2) models were also assessed.

For opioid dispensing and buprenorphine utilization separately, our models were defined as:

$$ \lambda =\ln \left(\mathrm{a}/\mathrm{n}\right)=\ln \left(\mathrm{a}\right)-\ln \left(\mathrm{n}\right)={\upbeta}_0+\left[{\upbeta}_1{\mathrm{X}}_1+\cdots +{\upbeta}_7{\mathrm{X}}_7\right]+\left[{\upbeta}_8{\mathrm{X}}_8\right]+\left[{\upbeta}_9{\mathrm{X}}_9\right]+\left[{\upbeta}_{10}{\mathrm{X}}_{10}\right]+\left[{\upbeta}_{11}{\mathrm{X}}_{11}\dots {\upbeta}_{13}{\mathrm{X}}_{13}\right]+\left[-\ln \left(\mathrm{n}\right)\right] $$

where λ was a rate of opioid dispensing or buprenorphine utilization per county-month residential population, *a* was a count of opioid dispensing or buprenorphine utilization, and β_0_ is the intercept.

X_1_, …_,_ X_7_ were the independent (exposure) variables that designate the presence or absence of the seven intervention strategies for any given month and county. Each strategy was represented as dichotomous variable with no implementation as the referent (coded 0), and implementation (coded 1).

X_8_ was the county-month rate of outpatient prescriptions dispensed for opioid analgesics in units of 1000, with total resident population as the denominator. This measure was only used as a covariate in models of buprenorphine utilization.

X_9_ is the county health status variable of linearly interpolated annual z-scores of Health Factors from RWJF County Health Rankings. This variable was used as a marker for general community health status and was included to control for potential confounding by changes to general community health status over time and between counties.

X_10_ was a variable for calendar year included to remove linear trends over time (“secular trend”).

X_11_, X_12_, and X_13_ were indicator variables for seasonality, implemented with indicator coding for spring, summer and fall, with winter as the referent. These variables are included to de-trend for seasonal effects on overdose and related outcomes, which were observed in preliminary data analysis. For opioid overdoses in 2010, a Walter and Elwood analysis of seasonality using the exact method test suggested the presence of seasonality (chi-square 5.8, *p* = 0.05, 2 df) with a peak in March.

The offset term ln(n) was the log_e_ denominator of the rates, defined as the resident population of the county. Annual population was obtained from the National Center for Health Statistics and linearly interpolated by month.

Goodness-of-fit was assessed using Akaike and Bayesian Information Criterion (AIC and BIC), with smaller values indicating better fit relative to an intercept-only model. We assessed over-dispersion using Deviance and Pearson’s chi-square divided by its degrees of freedom. Initially, univariate models were used to examine each of the individual strategies, without any adjustment for trends by year and season, county health status, or other strategies. Multivariable adjusted models examined the associations for each of the seven strategies while controlling for the other six strategies, with additional adjustment for year and season, and county health factors. Adjusted models of buprenorphine utilization also included adjustment for each county’s population-based rate of OA prescribing.

## Results

A total of 74 out of 100 NC counties implemented any strategy of PL by the end of the intervention period, covering 70% of the state population. Non-implementing counties were either ineligible due to a lack of resources or did not submit a funding application.

Over the 2009–2014 study period, unique annual OA patients decreased by 6.9%, from 23.0% of all state residents in 2009 to 21.4% in 2014 (Alexandridis et al., 2018). Annual prescriptions dispensed for OA increased by 17.3%, from 6.22 million to 7.30 million, an 11.4% increase (0.66 to 0.73 per person-year). The most commonly dispensed OAs were hydrocodone, oxycodone, codeine and morphine.

### Opioid analgesic dispensing

In univariate models (no adjustment), we found weak associations between the adoption of PL strategies and the rate of OA prescription dispensing (Table 2). MAT expansion was associated with a 16% increase in OA dispensing (IRR: 1.16; 95% CI: 1.11, 1.20), and Diversion Control efforts were associated with a 15% increase (IRR: 1.15; 95% CI: 1.12, 1.17) in OA dispensing.

**Table 2:** Associations between Project Lazarus implementation and opioid analgesic prescribing, by strategy, North Carolina, 2009–2014

				Univariate Models^a^			Multivariable Adjusted Model^b^		
Strategy	Implementation Level	County-months	Rx OAs dispensed	IRR	95% CI	CLR	IRR	95% CI	CLR
Diversion Control	None	4971	23,512,894	1 (ref.)			1 (ref.)		
	Any	2229	17,848,868	1.15	1.12, 1.17	1.044	1.06	1.03, 1.09	1.056
Naloxone Policies	None	6216	34,103,605	1 (ref.)			1 (ref.)		
	Any	984	7,258,157	1.08	1.06, 1.11	1.038	0.97	0.95, 0.99	1.048
Community Education	None	5969	30,353,079	1 (ref.)			1 (ref.)		
	Any	1231	11,008,683	1.11	1.09, 1.13	1.042	1.00	0.97, 1.03	1.058
Provider Education	None	4962	25,608,213	1 (ref.)			1 (ref.)		
	Any	2238	15,753,549	1.13	1.10, 1.15	1.042	1.00	0.97, 1.03	1.060
Support for Patients with Pain	None	6684	34,023,002	1 (ref.)			1 (ref.)		
	Any	516	7,338,760	1.08	1.06, 1.10	1.029	0.96	0.93, 1.00	1.069
Hospital ED Policy	None	5485	29,009,970	1 (ref.)			1 (ref.)		
	Any	1715	12,351,792	1.11	1.08, 1.13	1.047	0.99	0.96, 1.01	1.055
Addiction Treatment	None	1559	13,442,436	1 (ref.)			1 (ref.)		
	Any	5641	27,919,326	1.16	1.11, 1.20	1.085	1.04	0.99, 1.08	1.090

*IRR* incidence rate ratio, *CI* confidence interval, *CLR* confidence limit ratio, *ED* Emergency Department

Results from Poisson GEE with county-month population offset

^a^Univariate unadjusted models for each strategy

^b^Adjusted multivariable model adjusts for the other six strategies, county health status, year and season

In fully-adjusted multivariable models accounting for implementation of all seven strategies, year and season, and county health status, these associations were attenuated. A statistically significant association between Diversion Control strategies and increased prescribing persisted (IRR: 1.06; 95% CI: 1.03, 1.09). No other strategy was associated with a 5% or higher increase or decrease in opioid prescribing. Notably, the strategy of Provider Education was not associated with any change in OA dispensing (IRR: 1.00; 95% CI: 0.97, 1.03).

### Buprenorphine utilization

In univariate models (no adjustment), each PL strategy was associated with a 54–82% increase in the rate of buprenorphine utilization (Table 3). After adjustment for time and season, these associations were greatly attenuated in single-strategy models; in fact, support programs for patients with pain were associated with a 15% *decrease* in buprenorphine (IRR: 0.85; 95% CI: 0.78, 0.93).

**Table 3:** Associations between Project Lazarus implementation and buprenorphine utilization, by strategy, North Carolina, 2009–2014

				Univariate Models^a^			Multivariable Adjusted Model^b^		
Strategy	Implementation Level	County-months	Monthly Bup. Patients	IRR	95% CI	CLR	IRR	95% CI	CLR
Diversion Control	None	4971	266,389	1 (ref.)			1 (ref.)		
	Any	2229	323,576	1.82	1.71, 1.93	1.128	1.00	0.93, 1.08	1.165
Naloxone Policies	None	6216	443,181	1 (ref.)			1 (ref.)		
	Any	984	146,784	1.67	1.57, 1.78	1.133	1.02	0.96, 1.08	1.122
Community Education	None	5969	379,482	1 (ref.)			1 (ref.)		
	Any	1231	210,483	1.68	1.59, 1.78	1.117	0.98	0.91, 1.05	1.164
Provider Education	None	4962	303,822	1 (ref.)			1 (ref.)		
	Any	2238	286,143	1.77	1.68, 1.87	1.117	1.00	0.93, 1.07	1.148
Support for Patients with Pain	None	6684	454,973	1 (ref.)			1 (ref.)		
	Any	516	134,992	1.54	1.45, 1.64	1.131	0.93	0.85, 1.02	1.200
Hospital ED Policy	None	5485	360,241	1 (ref.)			1 (ref.)		
	Any	1715	229,724	1.68	1.57, 1.80	1.146	0.98	0.92, 1.04	1.134
Addiction Treatment (Policy Only)	None	6549	512,646	1 (ref.)			1 (ref.)		
	Any	651	77,319	1.62	1.51, 1.74	1.148	0.98	0.91, 1.06	1.161

*IRR* incidence rate ratio, *CI* confidence interval, *CLR* confidence limit ratio, *ED* Emergency Department

Results from Poisson GEE with county-month population offset

^a^Univariate unadjusted models for each strategy

^b^Adjusted multivariable model adjusts for the other six strategies, county opioid analgesic prescribing rate, county health status, year and season

In fully-adjusted multivariable models including all seven PL strategies, only support programs for patients with pain were associated with a change of 5% or greater in buprenorphine use (IRR: 0.93; 95% CI: 0.85, 1.02), and no strategy was associated with a statistically significant change. The addiction treatment strategy hypothesized to have a direct impact on this outcome was associated with a 2% reduction in buprenorphine use (IRR: 0.98; 95% CI: 0.91, 1.06). An additional model of only the addiction treatment strategy (including adjustment for county health status in addition to year and season, but without the other six strategies) found no association (IRR: 1.00; 95% CI: 0.92, 1.09).

## Discussion

Project Lazarus was implemented statewide in NC as a community-based program with multi-agency support. Its goals were to address opioid supply, demand, and harm reduction. PL sought to improve access to MAT and reduce opioid prescribing, while maintaining legitimate access to opioids for patients with chronic pain. The results of this analysis, together with our previous analysis of the association between PL and overdose morbidity and mortality, indicate that implementation of the PL strategies neither appreciably reduced opioid dispensing nor increased buprenorphine utilization (Alexandridis et al., 2018).

For a community-coalition-based program such as PL to be successful, as was observed in the pilot implementation in Wilkes County, NC, a strong community-public health partnership needs to be established (Albert et al., 2011). Indicators of a strong partnership include sustained and focused engagement by a local health department or similar public health agency with health care provider networks and/or key enforcement agencies, such as local law enforcement (Alexandridis et al., 2017). Such partnerships are relatively uncommon in communities, particularly around the issue of substance use, pain, opioids, or overdose. Local Health Departments offer a potential starting point for a coalition to crystalize around, but deep engagement with stakeholders outside of the local public health infrastructure is also critical (Alexandridis et al., 2017). The maximal annual funding, less than $35,000, provided to the PL coalitions was insufficient for the hiring of full-time community health worker organizers with sufficient budgets for implementation activity. Even if funding were sufficient to hire full-time employees, the motivation for various activities must also be internal to the community to achieve the greatest sustained effect. It is possible that we may have seen a greater effect from the statewide PL program if both funding levels and community readiness to implement actions based on the PL model had been at higher levels.

### Opioid prescribing

Diversion control efforts were the lone PL strategy associated with a statistically significant, 6% increase in opioid dispensing. Though unanticipated in its direction, this association was not clinically significant in its magnitude. Given the consistently high reported levels of unused controlled substances (CS) sharing between friends and family as reported by national data (Lipari & Hughes, 2017), one possible explanation is that aggressive take-back and drop-box efforts have led to modest increases in people seeking opioid prescriptions (Lewis et al., 2014; Wakeland et al., 2015). Likewise, other forms of anti-diversion law enforcement activity may have led to increases in the seeking of legitimate opioid prescriptions. It is also possible that this result is due to bias resulting from a misclassification of exposure in law enforcement trainings, which were a component of the diversion control strategy. The SBI targeted known areas of high opioid diversion activity for their trainings, which were in turn attended by law enforcement officers from multiple counties. As we were only able to capture the counties where trainings occurred, it is possible that counties were uncredited for the implementation of this strategy.

A previous study in Massachusetts demonstrated a significant decline in opioid prescribing and unique opioid patients after a comprehensive opioid and pain policy was adopted by a large statewide private insurer (Garcia et al., 2016). Our null result highlights potential limitations of diffuse community coalitions to create significant changes in prescriber practice as compared to a centralized, insurance-directed approach. The lack of impact of PL statewide implementation on prescribing may potentially reflect insufficient investment in local coalition activities. Additionally, it is important to note that PL, as implemented statewide in NC, was not designed with an explicit focus on reducing opioid dispensing volumes, but rather promoting appropriate pain management. The community-facing supply reduction efforts of PL focused on the prevention of prescription opioid sharing through unused drug disposal and education, whereas the healthcare-facing efforts addressed acute opioid prescribing in EDs and chronic pain treatment among community-based physicians. Only these latter physician education strategies would be expected to have a direct effect on opioid prescribing; however, a reduction was not observed in this study.

It is also likely that the effectiveness of PL activities to limit prescribing were affected by the changing pace and form of the overdose epidemic in the US during the implementation period. When PL was initially piloted in Wilkes County, NC, through the planning of the statewide implementation, it was not anticipated that nested epidemics of heroin and fentanyl overdose would occur at the scale since documented (Ciccarone, 2017; Unick et al., 2013; Cicero et al., 2015). At the time of the implementation, there was evidence that an inflection point in the epidemic had been reached (Dart et al., 2015a; Dart et al., 2015b). Future community-based efforts to reduce overdose must have the capacity to respond rapidly to evolving patterns of substance use and develop *a priori* contingency plans. One potential tool that state or federal agencies could use to identify motivated communities is the Community Readiness scale developed by the Tri-Ethnic Center for Prevention Research (Ringwalt et al., 2018). A multi-stage process could first identify communities with high motivation and infrastructure to deploy a community-based program, and target them to implement a PL-like program, while simultaneously developing motivation and infrastructure using other approaches elsewhere.

### Buprenorphine utilization

We found no strong association between any component of PL and buprenorphine utilization in adjusted models. Unadjusted univariate models indicated consistent increases in the utilization rate, even for strategies not expected to have a direct impact on buprenorphine, which were hypothesized to be the result of secular trends in MAT over the study period.

In our previous study, we found the PL addiction treatment strategy was associated with increased overdose mortality (Alexandridis et al., 2018). Together with the findings presented here, this suggests that areas with high MAT utilization were not necessarily influenced by PL, as PL-related MAT policy changes were not associated with a change in the rate of buprenorphine utilization. We focused on buprenorphine specifically because of its advantages in the management of opioid use disorder in rural areas (Kraus et al., 2011), and because buprenorphine’s non-MAT use remains closely linked to the clinical management of patients with high risks of opioid dependence or use disorder (Fiellin et al., 2014; Blondell et al., 2010).

It is important to note that even buprenorphine MAT requires a substantial investment to reduce fatal overdose. National surveillance of buprenorphine and heroin overdose in France, where buprenorphine accounts for well over 80% of all MAT, found an 82% reduction in heroin overdose deaths between 1995 and 2003 after the introduction of community-based MAT through primary care providers and community pharmacies in 1996 (Emmanuelli & Desenclos, 2005; Carrieri et al., 2006). However, total MAT utilization increased 100-fold nationally in that time period, and each prevented death was associated with upwards of 200 MAT patients. Within the US, the training requirement and patient limits imposed by the Drug Addiction Treatment Act of 2000 provide additional challenges to the effective implementation of buprenorphine-based MAT, due to incomplete coverage of MAT costs among Medicare/Medicaid patients (Knudsen et al., 2011). People in the custody of the criminal justice system also face considerable restrictions on access to MAT, particularly effective agonist-based MAT, as its use is mediated by drug court staff, judges, correctional facilities, and local and state politics (Friedmann et al., 2012; Brinkley-Rubinstein et al., 2017). These challenges and barriers to treatment underscore the difficulties faced in moving beyond simple supply reduction approaches to community-based addiction treatment (Dasgupta et al., 2018).

Recent strategy recommendations, such as the President’s Commission Report, have heavily stressed substance abuse treatment expansion, particularly maintaining access to MAT (Christie et al., 2017). Highly motivated and effective community coalitions are only able to expand or maintain such MAT programs when they are supported or endorsed by diverse federal and state entities, such as Medicaid/Medicare; justice departments, drug courts, and correctional systems; and SAMHSA and its state-level counterparts. Stigma and resistance to agonist MAT affects all levels of this structure, and coalition activity may have a limited impact on local attitudes and views (Ringwalt et al., 2018). Future studies should analyze the uptake of MAT using a more expansive view of these institutional barriers, treating coalition and community activity with regards to MAT as an effect modifier of state and federal policies.

## Limitations

Our evaluation of PL was limited by funder priorities that all North Carolina counties should implement PL, necessitating an observational interrupted time series study design. We were therefore unable to randomize communities to receive PL funding and supports, and residual or uncontrolled confounding may be present. The associations between PL strategies and our outcomes cannot be interpreted as causal. In particular, we were unable to quantify any of the selection factors associated with higher intensity of PL implementation in a given community. Multiple factors influencing coalition activity and substance use could not be obtained with the appropriate spatial and temporal resolution, including: previous collaborations among stakeholders (Kegler et al., 2010), external measures of coalition leadership (Kegler et al., 1998), private insurance policy changes (Garcia et al., 2016), prescriber utilization of the PDMP (Delcher et al., 2015), and the implementation of the Risk Evaluation and Mitigation Strategies (REMS) for transmucosal immediate-release fentanyl and extended-release/long-acting opioid analgesics (Food and Drug Administration, 2012; Cepeda et al., 2017). All were therefore assumed to have nondifferential effects. Because the REMS were not fully implemented by the end of the intervention period their likely effect was minimal. Our model of the intervention also assumes that implementation of PL strategies occurs with high fidelity and that all the PL strategies have a sustained ongoing effect, or are continuously implemented. These are strong assumptions to make in the context of community coalition-based programs funded at relatively modest levels. Finally, although we did not detect changes in overall volume of prescribing, it is possible that the nature of prescribing was altered and inappropriate prescribing was reduced.

Outcomes for both analyses in this study were derived from PDMP data, which have the typical caveats of administrative, secondary data. Our ability to identify unique buprenorphine patients is limited by the proprietary entity resolution algorithms used to link prescriptions based on name, address, and date of birth, potentially more challenging in vulnerable populations that may be more geographically mobile (Galea & Vlahov, 2002). Unlinked records would result in overestimations of unique patients; we addressed this possible source of bias by using a prevalent rather than incident patient outcome.

Finally, our post-intervention period was limited to 22 months. The original pilot of PL in Wilkes County saw its greatest effect after three years of implementation (Albert et al., 2011). Future evaluations of community-based approaches to overdose should consider the length of the intervention and follow-up period.

## Conclusions

Despite other accomplishments, the statewide implementation of Project Lazarus in North Carolina did not meet its objectives of marked increases in the utilization of buprenorphine or reductions in opioid analgesic prescribing. Future support for community coalitions addressing the opioid crisis may need a more narrow focus and targeted coalition capacity building to ensure impacts on such outcomes as prescribing behaviors and addiction treatment.

## Abbreviations

CCNC: Community Care of North Carolina
CSRS: North Carolina controlled substance reporting system
ED: Emergency Department
GEE: Generalized estimating equations
IRR: Incidence rate ratios
MAHEC: Mountain Area Health Education Center
MAT: Medication assisted treatment
MOR: Mu-opioid receptor
NC DHHS: North Carolina Department of Health and Human Services
NC: North Carolina
NC-TOPPS: North Carolina treatment outcomes and program performance system
OA: Opioid analgesics
ONDCP: Office of National Drug Control Policy
PDMP: Prescription Drug Monitoring Program
PL: Project Lazarus
SAMHSA: Substance Abuse and Mental Health Services Administration
SBI: North Carolina State Bureau of Investigation
